# Mental Health in Multiple Sclerosis During the COVID-19 Outbreak: A Delicate Balance between Fear of Contagion and Resilience

**DOI:** 10.1007/s10880-022-09849-w

**Published:** 2022-01-22

**Authors:** Laura Rosa, Cristiano Scandurra, Alessandro Chiodi, Maria Petracca, Teresa Costabile, Francesca Lauro, Marcello Moccia, Antonio Carotenuto, Nelson Mauro Maldonato, Vincenzo Brescia Morra, Roberta Lanzillo

**Affiliations:** 1 InterUniversity Center for Research in Neurosciences (CIRN), University of Campania “Luigi Vanvitelli”, Napoli, Italy; 2 Department of Neurosciences and Reproductive and Odontostomatological Sciences, University of Naples Federico II, Napoli, Italy; 3 Intradepartmental Program of Clinical Psychology, Federico II University Hospital, Naples, Italy; 4 Department of Human Neurosciences, Sapienza University, Rome, Italy

**Keywords:** Multiple sclerosis, Disability, Illness perception, Resilience, COVID-19, Mental health

## Abstract

The current study aimed at exploring the relationship between objective disability, illness perceptions, resilience, fear of COVID-19, and psychological distress (i.e., anxiety, depression, and stress) in people with multiple sclerosis (pwMS) during the second wave of the COVID-19 outbreak. A group of 122 pwMS recruited in an Italian university hospital took part in this cross-sectional monocentric study. Hierarchical multiple linear regression analyses were performed to assess the strength of the hypothesized associations. Results indicated that, differently from cognitive impairment, motor disability was positively associated with anxiety. However, accounting for subjective illness perception, such association was no longer significant. Moreover, accounting for both protective and risk factors in the models, even illness perception was no longer significant, highlighting the central role of resilience and fear of COVID-19 in explaining the negative emotional outcomes. Implications for clinical interventions and psychoeducational trainings are discussed.

## Introduction

Multiple sclerosis (MS) is a chronic disabling disease which affects both the motor and cognitive systems, exerting a dramatic impact on mental health (Chwastiak & Ehde, 2007). Indeed, approximately 25–50% of people with MS (pwMS) develop a form of major depression over the course of their lives (Feinstein et al., 2014). Disease severity represents the most robust depression-correlated dimension (Chwastiak et al., 2002), with people reporting moderate or severe disability being at higher risk to report depressive symptoms (Bamer et al., 2008). Cognitive decline—that occurs in 40–65% of cases (Bobholz & Rao, 2003; McIntosh-Michaelis et al., 1991; Rao et al., 1991)—also affects mental health and pwMS showing impaired cognition present a higher prevalence of psychiatric symptoms than those with a similar degree of disability and duration of illness but with intact cognitive function (Khader et al., 2019).

Beyond objective disability, illness perception (i.e., cognitive and emotional representations of one’s own illness) also plays a significant role in psychological distress of pwMS (Benyamini, 2011), independently affecting their quality of life (QoL) (Spain et al., 2007).

Luckily, objective disability and illness perception are not the only factors influencing psychological distress in MS. Indeed, people diagnosed with chronic diseases tend to develop resistance strategies, adaptively responding to the challenges caused by the disease itself (de Ridder et al., 2008). Resilience, which can be defined as the ability to adapt to and bounce back from adversity (Zimmerman, 2013), is one way to cope with the stress caused by chronic diseases and represents a fundamental protective factor for mental distress in pwMS, where it acts as a buffer, decreasing the negative effects of affective disorders on QoL (Rainone et al., 2017).

Recently, in the scenario of the Covid-19 outbreak, the balance between the above-mentioned risk and protective factors has been further complicated by the emergence of new elements (Kontoangelos et al., 2020). Indeed, beyond the evidence that COVID-19 pandemic has increased levels of anxiety, depression, and stress in the general population (Lima et al., 2020; Maldonato et al., 2020), other specific and significant emotional reactions have been found, such as fear of being infected by the severe acute respiratory syndrome coronavirus 2 (SARSCoV-2) (Ahorsu et al., 2020; Rubin & Wessely, 2020; Shigemura et al., 2020). Fear may be conceptualized as an unpleasant emotion associated with emotional avoidance concerning specific stimuli. Some specific aspects of the COVID-19 pandemic seem to have exacerbated feelings of fear among the population, such as uncertainty about its spreading and evolution and the initial absence of vaccines (Ornell et al., 2020). While moderate levels of fear of COVID-19 may be helpful as they lead people to engage in protective behaviors, extreme levels of fear of COVID-19 may become detrimental to psychological health, as people may not think rationally when reacting to COVID-19 (Bochicchio et al., 2021; Garfin et al., 2020; Scandurra et al., 2021; Sloan et al., 2021). Previous studies demonstrated that the fear of COVID-19 is a significant predictor of depression, anxiety, and stress (Rodríguez-Hidalgo et al., 2020; Yıldırım et al., 2020) and it is plausible to hypothesize that this may be true even in pwMS, who perceive themselves as most at risk (Borriello & Ianniello, 2020; Foerch et al., 2020; Herman et al., 2020; Salari et al., 2020).

Previous studies tackling the issue of psychological distress in pwMS during the Covid-19 outbreak have reported how the main concerns of pwMS were the possibility of MS worsening in case of infection (36.4%), the potential difficulties in treatment availability (43.6%), and the impossibility to attend hospital visits as usual (72.4%) (Stojanov et al., 2020). As mental distress goes, the only significant change reported so far relates to depression (Chiaravalloti et al., 2020; Costabile et al., 2020) but, surprisingly, it was either reported to be independent from disability (Chiaravalloti et al., 2020) or only indirectly driven by disability via facilitation by a passive attitude (Costabile et al., 2020). Although it was previously hypothesized that this unexpected lack of correlation between disability and psychological distress could be the result of the contingent pandemic situation (Costabile et al., 2020), researchers did not formally explore the effect of the fear of Covid-19. Additionally, previous investigations focused on objective disability (assessed either via patient reported outcomes or neurological/cognitive examination) (Chiaravalloti et al., 2020; Costabile et al., 2020), but no study considered independently the role of “subjective” disability. Here, to fill these gaps and to achieve a more complete characterization of mental distress in MS at the time of the pandemic, we specifically investigated the impact of the fear of COVID-19 on psychological distress and verified how the relationship between known risk factors (objective motor and cognitive disability, subjective disability expressed by patients’ illness perception), protective factors (resilience), and mental distress is affected by the presence of this new contingent stressor.

## Methods

### Procedures and Participants

This is a cross-sectional study conducted as a web survey. PwMS who had access to the MS center of the University of *Naples Federico II* between September and October 2020 (a period in which in Italy the Covid-19 contagion curve began to rise steeply) were asked to take part in this study and were provided the link to the survey, which was uploaded on *EUsurvey*. By clicking on the link provided, participants were directed to the first page of the survey, containing the informed consent of the study, objectives, risks and benefits, and information about the proposing researchers. In the informed consent it was clearly reported that the data would have been analyzed in aggregate form and the results published in scientific journals. After reading all information, participants gave their consent to participate in the survey by clicking “I accept to take part in the survey.”

To avoid missing data, all questions had to be completed in order to proceed through the survey that could be submitted only if all fields had been filled. Eligibility criteria were as follows: (a) being at least 18 years old and (b) being able to understand the informed consent. A total of 122 participants took part in the survey.

The current study was approved by the Ethical Committee of the University of Naples Federico II (protocol number 160/20/ES1), designed in the respect of principles of the Declaration of Helsinki and in accordance with the Strobe Statement, and conducted following the EU General Data Protection Regulation (GDPR).

### Measures

#### Objective Measures of Disability

To assess objective disability, a neurological examination with expanded disability status scale (EDSS) scoring was performed on the same day of the questionnaire completion, while the symbol digit modalities test (SDMT) was retrieved from the database collecting neuropsychological scores administered yearly for clinical practice. The EDSS is the most widely used outcome measure to assess MS-related disability in clinical trials (Uitdehaag, 2018). It is an ordinal scale ranging from 0 (normal neurological exam) to 10 (death due to MS) based on the severity of findings on the neurological evaluation of eight functional systems, walking ability, and ability to carry out activities of daily living, with higher scores indicating worse disability (Kurtzke, 1983). SDMT is a test of cognitive processing speed and sustained attention (Benedict et al., 2017) which has been recommended as screening tool for cognitive impairment in MS (Kalb et al., 2018), cognitive monitoring in clinical practice, and cognitive assessment in research (Sumowski et al., 2018). Higher scores indicate better cognitive performance.

#### Subjective Measure of Disability

To assess the subjective perception of one’s MS, we used the brief form of the brief illness perception questionnaire (BIPQ; Broadbent et al., 2006), an 8-item scale assessing the cognitive and emotional representations of illness on an 11-point Likert scale. Higher scores indicate negative subjective perception, reflecting a higher perceived threat.

#### Resilience

Resilience was assessed through the resilience scale (RS; Wagnild & Young, 1993), a 10-item questionnaire measuring resilience as a personal characteristic helping to buffer the negative effects of stress and promoting adjustment. Response options range from 1 (“strongly disagree”) to 7 (“strongly agree”), with higher scores reflecting higher levels of resilience.

#### Fear of COVID-19

The fear of COVID-19 was measured through the Fear of COVID-19 scale (FCV-19S; Ahorsu et al., 2020), a 7-item scale measuring the specific fear associated with the risk of being infected by SARSCoV-2 on a 5-point Likert scale, from 1 (“strongly disagree”) to 5 (“strongly agree”). Higher scores indicate higher levels of fear of contracting SARSCoV-2.

#### Psychological Distress

Psychological distress was assessed through the depression anxiety stress scales (DASS-21; Lovibond & Lovibond, 1995), a 21-item scale measuring negative emotional states along the 3 axes of depression, anxiety, and stress on a 4-point Likert scale, from 0 (“Did not apply to me at all—Never”) to 3 (“Applied to me very much or most of the time—Almost always”). Specifically, the Depression scale assesses dysphoria, hopelessness, devaluation of life, self-deprecation, anhedonia, lack of interest, and inertia; the Anxiety scale evaluates autonomic arousal, skeletal muscle effects, and subjective experience of anxious affect; and the Stress scale measures difficulty relaxing, nervous arousal, and being easily upset, irritable, and impatient. Higher scores on each subscale indicate higher psychological distress.

### Statistical Analyses

All statistical analyses were performed using SPSS version 26, setting the level of significance at 0.05.

We first analyzed participants characteristics through descriptive statistics (distribution of frequencies, means, and standard deviation) and bivariate correlations between variables.

Then, we performed three hierarchical multiple linear regression analyses, based on the three DASS-21 subscales (i.e., depression, anxiety, and stress) as dependent variables. In these models, demographics were entered in step 1 as covariates, objective disability factors in step 2, people’s illness perception in step 3, and resilience and fear of COVID-19 in step 4. Cohen’s *f2* method was used as an indicator of the effect size, with *f*^*2*^ ≥ 0.02, *f*^*2*^ ≥ 0.15, and *f*^*2*^ ≥ 0.35 representing small, medium, and large effect sizes, respectively (Cohen, 1988).

To avoid problems of multicollinearity, all linear variables included in the regression models were mean centered and variance inflation factor (VIF) was calculated as a measure of the estimated regression coefficients increase in variance related to predictor’s intercorrelation. Conventionally, VIFs near or above 5 are accepted (Akinwande et al., 2015). Finally, as most of the enrolled patients were under second-line therapies for MS, we conducted a post hoc analysis to assess the potential role of therapy on the fear of COVID-19, evaluating mean differences between pwMS assuming first-line drugs and those assuming second-line drugs through a Student’s t test.

## Results

### Participants Characteristics

Participants ranged in age from 18 to 70 (*M* = 37.7; *SD* = 11.97). As regards gender identity, 39 (32%) were identified as men and 83 (68%) were identified as women. Time from diagnosis ranged from 10 months to 33 years (*M* = 11.41; *SD* = 7.93). Most of the participants were married (*n* = 59; 48.4%) or single (*n* = 56; 45.9%), while only 2 (1.6%) were divorced and 5 (4.1%) cohabitants. Furthermore, 34 (27.9%) of pwMS had a personal knowledge of someone infected by the SARSCoV-2, while 16 (13.1%) knew someone dead for SARSCoV-2. No participants resulted infected by SARSCoV-2 at the time of the study nor had been infected in the past. Finally, 23.5% of participants assumed first-line drugs (i.e., interferon-beta 1a, glatiramer acetate, dimethyl fumarate), while the remaining were treated with second-line drugs (i.e., fingolimod, siponimod, natalizumab, ocrelizumab, rituximab, alemtuzumab, cladribine).

### Descriptive Statistics and Bivariate Correlations

Means, standard deviations, and bivariate correlations between all variables are reported in Table 1.

**Table 1:** Bivariate correlations between motor and cognitive disability status, illness perceptions, fear of COVID-19, resilience, and mental heal

	1	2	3	4	5	6	7	8	M ± SD or Mdn (range)
1. EDSS	–								2.50 (1.00–7.50)
2. SDMT	− .29**	–							49.39 ± 16.19
3. Illness perception	.55***	− 0.15	–						5.01 ± 1.51
4. Fear of COVID-19	0.13	0.05	.37***	–					2.43 ± .77
5. Resilience	− .30**	.27**	− .52***	− .21*	–				5.51 ± .87
6. DASS—depression	.22*	− 0.14	.42***	.33***	− .70***	–			7.27 ± 4.65
7. DASS—anxiety	0.16	− 0.06	.43***	.51***	− .37***	.67***	–		4.06 ± 3.75
8. DASS—stress	0.11	0.07	.35***	.42***	− .47***	.83***	.75***	–	5.35 ± 4.93

*EDSS* expanded disability status scale, *SDMT* symbol digit modalities test, *DASS* depression anxiety stress scales, *M* mean, *SD* standard deviation, *Mdn* median

*p < .05, **p < .01, ***p < .001

As regards the objective disability factors, the EDSS correlated positively with illness perception and depression and negatively with SDMT and resilience, while SDMT correlated negatively only with resilience. Resilience scores were higher in participants showing better cognitive performance. Illness perception correlated positively with fear of COVID-19, depression, anxiety, and stress and negatively with resilience. Fear of COVID-19 resulted negatively correlated with resilience and positively correlated with depression, anxiety, and stress.

All measures showed an adequate internal consistency reliability, ranging from 0.72 to 0.92.

Fear of COVID-19 was significantly lower in pwMS assuming first-line drugs than in pwMS assuming second-line drugs (*t* = 2.20; *p* = .03).

### Associations Between Objective Disability, Illness Perceptions, Fear of COVID-19, Resilience, and Psychological Distress

Results for regressions of all negative emotional outcomes (i.e., depression, anxiety, and stress) on objective disability, people’s illness perceptions, resilience, and fear of COVID-19 are reported in Table 2. All VIFs were acceptable, ranging from 1.06 to 2.12 in all models tested.

**Table 2:** Multiple linear regressions of mental health on objective disability status, illness perceptions, resilience, and fear of COVID-19

	Depression			Anxiety			Stress		
	*B* (SE)	95% CI	*β*	*B* (SE)	95% CI	*β*	*B* (SE)	95% CI	*β*
*Step 1—control variables*									
Age	0.04 (0.05)	− 0.06, 0.13	0.09	− 0.02 (0.04)	− 0.11, 0.05	− 0.08	− 0.03 (0.05)	− 0.13, 0.06	− 0.09
Gender (man)	− 0.62 (1.14)	− 2.89, 1.65	− 0.06	0.37 (0.94)	− 1.49, 2.25	0.04	− 0.66 (1.09)	− 0.13, 0.06	− 0.07
Time of diagnosis	− 0.03 (0.08)	− 0.18, 0.13	− 0.04	0.01 (0.06)	− 0.11, 0.14	0.03	− 0.05 (0.07)	− 2.84, 1.53	− 0.08
Knowledge of COVID-19 infected	− 0.77 (1.32)	− 3.39, 1.85	− 0.07	− 1.15 (1.09)	− 3.32, 1.01	− 0.13	0.36 (1.27)	− 0.20, 0.10	0.04
Knowledge of COVID-19 dead	0.45 (1.72)	− 2.97, 3.87	0.03	0.75 (1.42)	− 2.07, 3.58	0.07	− 0.62 (1.66)	− 2.16, 2.88	− 0.05
	*R*^*2*^ = .01; *F* = 0.21			*R*^*2*^ = .02; *F* = 0.36			*R*^*2*^ = .03; *F* = 0.44		
*Step 2—objective disability*									
Age	− 0.02 (0.05)	− 0.13, 0.09	− 0.06	− 0.08 (0.04)	− 0.17, 0.01	− 0.24	− 0.06 (0.05)	− 0.16, 0.05	− 0.15
Gender (man)	− 0.41 (1.14)	− 2.67, 1.86	− 0.04	0.72 (0.92)	− 1.12, 2.26	0.08	− 0.37 (1.11)	− 2.59, 1.84	− 0.04
Time of diagnosis	− 0.02 (0.08)	− 0.17, 0.13	− 0.04	0.01 (0.06)	− 0.11, 0.14	0.03	− 0.05 (.07)	− 0.20, 0.09	− 0.09
Knowledge of COVID-19 infected	− 1.03 (1.34)	− 3.69, 1.63	− 0.10	− 1.65 (1.08)	− 3.81, 0.51	− 0.19	− 0.09 (1.31)	− 2.69, 2.51	− 0.01
Knowledge of COVID-19 dead	0.53 (1.67)	− 2.85, 3.90	0.04	0.74 (1.37)	− 1.99, 3.48	0.06	− 0.69 (1.66)	− 3.99, 2.61	− 0.05
EDSS	0.75 (0.42)	− 0.08, 1.58	0.23	0.91 (0.34)	0.23, 1.58	.34**	0.61 (0.41)	− 0.20, 1.42	− 0.19
SDMT	− 0.03 (0.03)	− 0.10, 0.04	− 0.11	− 0.01 (0.03)	− 0.06, 0.05	− 0.03	0.02 (0.03)	− 0.05, 0.09	0.06
	*R*^*2*^ = .07; *ΔR*^*2*^ = .06; *F* = 0.86			*R*^*2*^ = .11; *ΔR*^*2*^ = .09*; *F* = 1.39			*R*^*2*^ = .05; *ΔR*^*2*^ = .03; *F* = 0.64		
*Step 3—illness perception*									
Age	− 0.04 (0.05)	− .14, 0.06	− 0.09	− 0.09 (0.04)	− 0.17, − 0.01	− 0.28	− 0.07 (0.05)	− 0.17, 0.03	− 0.19
Gender (man)	− 0.34 (1.05)	− 2.42, 1.75	− 0.03	0.77 (0.84)	− 0.90, 2.45	0.09	− 0.32 (1.05)	− 2.40, 1.77	− 0.03
Time of diagnosis	0.01 (0.07)	− .13, 0.15	0.01	0.04 (0.06)	− 0.07, 0.15	0.08	− 0.03 (0.07)	− 0.17, 0.11	− 0.04
Knowledge of COVID-19 infected	− 0.14 (1.25)	− 2.63, 2.34	− 0.01	− 0.89 (1.00)	− 2.90, 1.10	− 0.10	0.67 (1.25)	− 1.81, 3.16	0.07
Knowledge of COVID-19 dead	− 0.13 (1.57)	− 3.25, 2.98	− 0.01	0.19 (1.26)	− 2.32, 2.70	0.02	− 1.25 (1.57)	− 4.37, 1.87	− 0.09
EDSS	− 0.09 (0.44)	− 0.97, 0.77	− 0.03	0.20 (0.35)	− 0.50, 0.90	0.07	− 0.11 (0.44)	− 0.98, 0.76	− 0.03
SDMT	− 0.04 (0.03)	− 0.10, 0.03	− 0.12	− 0.10 (0.03)	− 0.06, 0.04	− 0.04	0.01 (0.03)	− 0.05, 0.08	0.05
Illness perception	0.19 (0.05)	0.09, 0.29	.47***	0.16 (0.04)	0.08, 0.24	.48***	0.16 (0.05)	0.07, 0.26	.41**
	*R*^*2*^ = .22; *ΔR*^*2*^ = .15***; *F* = 2.89**			*R*^*2*^ = .27; *ΔR*^*2*^ = .16***; *F* = 3.65**			*R*^*2*^ = .17; *ΔR*^*2*^ = .12**; *F* = 2.07*		
*Step 4—risk and protective factors*									
Age	− 0.03 (0.04)	− 0.11. 0.04	− 0.09	− 0.07 (0.04)	− 0.14, 0.01	− 0.22	− 0.05 (0.04)	− 0.14, 0.03	− 0.14
Gender (man)	− 0.63 (0.85)	− 2.32, 1.06	− 0.06	− 0.02 (0.80)	− 1.62, 1.57	− 0.01	− 1.07 (0.94)	− 2.94, 0.81	− 0.11
Time of diagnosis	− 0.03 (0.06)	− 0.14, 0.09	− 0.04	− 0.01 (0.05)	− 0.12, 0.09	− 0.02	− 0.08 (0.06)	− 0.21, 0.04	− 0.14
Knowledge of COVID-19 infected	− 0.59 (0.99)	− 2.55, 1.37	− 0.06	− 1.42 (0.93)	− 3.27, 0.43	− 0.16	0.05 (1.09)	− 2.13, 2.22	0.05
Knowledge of COVID-19 dead	0.31 (1.23)	− 2.13, 2.75	0.02	0.44 (1.16)	− 1.86, 2.75	0.04	− 0.83 (1.36)	− 3.54, 1.87	− 0.06
EDSS	− 0.08 (0.34)	− 0.60, 0.76	0.02	0.33 (0.32)	− 0.31, 0.98	0.12	0.08 (0.38)	− 0.67, 0.84	0.03
SDMT	− 0.01 (0.03)	− 0.06, 0.05	− 0.01	− 0.02 (0.02)	− 0.06, 0.03	− 0.06	0.03 (0.03)	− 0.03, 0.08	0.09
Illness perception	.02 (.04)	− .07, .11	0.05	0.07 (0.04)	− 001, 0.16	0.22	0.01 (0.05)	− 0.09, 0.11	0.02
Resilience	− 3.42 (0.51)	− 4.44, − 2.40	− .62***	− 0.44 (0.48)	− 1.41, 0.52	− .09	− 2.08 (0.57)	− 3.21, − 0.95	− 0.39***
Fear of COVID-19	0.18 (0.08)	0.01, 0.35	0.20*	0.32 (.08)	0.15, 0.47	.42***	0.36 (0.10)	0.14, 0.51	0.41**
	*R*^*2*^ = .54; *ΔR*^*2*^ = .31***; *F* = 9.05***			*R*^*2*^ = .40; *ΔR*^*2*^ = .13***; *F* = 5.19**			*R*^*2*^ = .39; *ΔR*^*2*^ = .22**; *F* = 5.06***		

*B* unstandardized regression coefficient, *SE* standard error, *CI* confidence interval, *β* unstandardized regression coefficient, *R*^*2*^ R-square, *ΔR*^*2*^ change in R^2^

***p < .001; **p < .01; *p < .05

Control variables were not associated with any of the negative emotional outcomes. Introducing objective disability factors in step 2 added significance only to anxiety (*f*^*2*^ = 0.12), explaining 11% of its variation. Specifically, EDSS increased the likelihood of reporting anxious symptoms.

Adding illness perception in step 3, instead, added significance to all regression models, explaining 22, 27, and 17% of the variation of depression, anxiety, and stress, respectively. Specifically, higher negative perception related to one’s own illness was associated with an increase in psychological distress, independently from objective disability. Additionally, the introduction of illness perception made EDSS no longer significant as a predictor of anxiety.

Instead, introducing resilience as a protective factor and fear of COVID-19 as a risk factor explained an additional 31, 13, and 22% of the variation of depression, anxiety, and stress, respectively, indicating that low levels of resilience increased the likelihood of reporting depressive and stress symptoms, but not anxious symptoms, while high levels of fear of COVID-19 increased the likelihood of reporting all negative emotional outcomes, i.e., depression, anxiety, and stress. The introduction of these factors made illness perception no longer significant as a predictor of all psychological distress variables.

The final statistical models accounted for 54, 40, and 39% of the variance in depression, anxiety, and stress, respectively, with large effect sizes (1.17, 0.66, and 0.64, respectively).

## Discussion

The current study assessed the role of objective disability, illness’ perception, resilience, and fear of COVID-19 in predicting psychological distress in a group of Italian pwMS recruited during the second wave of COVID-19 pandemic. Results indicated that, considering all variables in a unique model, the most significant predictive factors of psychological distress were resilience and fear of being infected by SARSCoV-2.

With regard to the role of objective disability in negative emotional outcomes, our findings showed that, differently from cognitive impairment, motor disability status was positively associated only with anxiety but that once the subjective illness perception was accounted for, such association was no longer significant. Thus, our results are not in line with previous studies finding associations between objective disability status and mental distress (Butler et al., 2016; Chwastiak & Ehde, 2007; Henry et al., 2019; Jones et al., 2014; Wood et al., 2013; Zorzon et al., 2001), while confirm previous research showing that people’s illness perceptions are better predictors of negative mental health outcomes than objective disability (Bassi et al., 2016; Jopson & Moss-Morris, 2003). Indeed, negative illness perceptions directly influence the individual’s emotional and cognitive response to the illness, thus representing a fundamental cognitive and emotional vulnerability factor which can be associated with psychological distress regardless of objective disability (Jopson & Moss-Morris, 2003).

However, our findings indicated that once both protective and risk factors were included in the models, even illness perception was no longer significant, highlighting the central role of both resilience and fear of COVID-19 in explaining, with large effect sizes, the psychological distress. This finding may be explained considering the historical moment during which participants answered the questions, corresponding to the second wave of COVID-19 pandemic. Indeed, regarding the fear of COVID-19, it is plausible to hypothesize, in line with Stojanov et al. (2020), that pwMS are strongly concerned about the possibility of being infected by the SARSCoV-2, as they perceive themselves as a vulnerable population. This might explain why the fear of COVID-19 is associated with negative emotional outcomes. Indeed, to date there is no scientific evidence that pwMS would experience higher risk than others if infected by SARSCoV-2. Thus, it is plausible to hypothesize that the fear of COVID-19 as a risk factor for psychological distress in pwMS would be related to implicit beliefs about the disease and the type of treatment rather than to objective conditions.

Interestingly, our findings have also shown that participants assuming second-line drugs, or rather those with a more aggressive/advanced disease status, show a higher fear of COVID-19 than those assuming first-line drugs. Indeed, as patients under treatment with second-line drugs have likely experienced a more aggressive disease course than those who are stable under treatment with first-line drugs, this might contribute to their self-perception of vulnerability. However, as not all second-line therapies expose pwMS to a greater infectious risk or more severe outcome (De Angelis et al., 2020; Thakolwiboon et al., 2020), we can infer that even in this case the risk perception is independent from the actual risk. This inference is supported by preliminary data concerning patients exposed to therapies potentially more predisposing to infection (i.e., cell depleting) (Parrotta et al., 2020; Safavi et al., 2020) that, contrary to these preliminary evidence, did not show a higher self-perception of vulnerability in our sample. However, the higher fear of COVID-19 showed by patients under second-line therapies, regardless from the drug-specific mechanism of action, may also be explained by the fact that most of these patients need to attend the hospital more frequently, given the administration schemes and monitoring requirements of second-line drugs. The necessity to frequently access the hospital during the COVID-19 outbreak could thus explain the greater concern and fear of contracting the virus in these individuals. Notwithstanding, our findings indicated that being resilient decreases the risk of reporting psychological distress, in particular depressive symptoms and stress. Thus, we confirm the role of resilience as one of the most significant protective factors in people with chronic diseases, in agreement with previous studies (American Psychological Association, 2020; de Ridder et al., 2008). Indeed, resilience ability may lead pwMS to better use their personal resources, adaptively responding to the challenges that the disease implies, thus resulting in a better QoL (Kasser & Zia, 2020; Ploughman et al., 2020; Rainone et al., 2017) and counteracting the development of psychological distress (Rainone et al., 2017).

Our findings, although interesting, should be considered in light of significant limitations. First, the cross-sectional nature of the study does not allow to make definitive and conclusive inferences about the causality of the relationships investigated. Second, the sample belongs to a single geographical area and, therefore, it is not fully representative of the population. International multicentric studies will be needed to overcome this limitation. Third, the enrollment procedure, based on recruiting patients attending the MS center, introduced a selection bias, with patients under second-line therapies being more apt to attend the center than those under first-line therapies. As a result, our findings are possibly more specific to patients with more aggressive disease course. Fourth, psychological distress was measured through explicit and self-report measures and thus answers may be influenced by subjective biases.

Despite these limitations, the present study has some significant implications for clinical practice. Indeed, our results suggests that, during the COVID-19 outbreak, clinical interventions should be mostly based on reinforcing resilience strategies and decreasing unjustified fear of being more prone to be infected by the SARSCoV-2, working through the emotional and cognitive dimensions related to such fear. For instance, within the resilience-based interventions, some previous studies have applied the principles of the acceptance and commitment therapy to pwMS, reporting significant improvements in resilience, QoL, depression, and stress (Alschuler et al., 2018; Halstead et al., 2020; Pakenham et al., 2018). This type of clinical intervention aims to increase psychological flexibility by working on aspects of acceptance and awareness. Similarly, mindfulness-based interventions may be also appropriate for pwMs, as trait mindfulness mediates the relationship between illness intrusiveness and depression in PwMS (Miller et al., 2020).

Considering that the fear of COVID-19 seems not linked to an objective risk, it would be impactful to implement psychoeducational training conducted by both physicians and psychologists, aimed at increasing people’s knowledge about MS and related therapies. Indeed, it is likely that having clinically correct information would decrease the fear of being infected and, consequently, the risk of developing psychological distress. Finally, the emotional side of the fear of COVID-19 may be addressed through short-term counseling interventions, focused on mental representations and emotions related to one’s condition of illness.

## Conclusion

This study highlights the crucial role of resilience and fear of COVID-19 in modulating the emergence of psychological distress in pwMS. Our findings suggest the need of implementing both clinical interventions and psychoeducational trainings, with the goal to increase resilience and decrease the fear of being infected by SARSCoV-2, in order to improve mental health status of pwMS in this historical moment.

## Data Availability

The data and materials that support the findings of this study are available from the corresponding author upon reasonable request.
